# Synthesis of Titanium Nitride Nanoparticles by Pulsed Laser Ablation in Different Aqueous and Organic Solutions

**DOI:** 10.3390/nano12101672

**Published:** 2022-05-13

**Authors:** Anton A. Popov, Gleb V. Tikhonowski, Pavel V. Shakhov, Elena A. Popova-Kuznetsova, Gleb I. Tselikov, Roman I. Romanov, Andrey M. Markeev, Sergey M. Klimentov, Andrei V. Kabashin

**Affiliations:** 1 National Research Nuclear University MEPhI, 115409 Moscow, Russia; gtikhonowski@gmail.com (G.V.T.); pvshakhov@mephi.ru (P.V.S.); eapopovakuznetsova@mephi.ru (E.A.P.-K.); limpo2003@mail.ru (R.I.R.); smklimentov@mephi.ru (S.M.K.); 2 Center for Photonics and 2D Materials, Moscow Institute of Physics and Technology, 141700 Dolgoprudny, Russia; celikov@physics.msu.ru (G.I.T.); markeev.am@mipt.ru (A.M.M.); 3 Laboratory LP3, Campus de Luminy, Aix-Marseille University, CNRS, 13288 Marseille, France

**Keywords:** pulsed laser ablation in liquids, laser ablation in aqueous and organic solutions, titanium nitride nanoparticles, XPS, EDX

## Abstract

Owing to a strong photothermal response in the near-IR spectral range and very low toxicity, titanium nitride (TiN) nanoparticles (NPs) synthesized by pulsed laser ablation in liquids (PLAL) present a novel appealing object for photo-induced therapy of cancer, but the properties of these NPs still require detailed investigation. Here, we have elaborated methods of femtosecond laser ablation from the TiN target in a variety of liquid solutions, including acetonitrile, dimethylformamide, acetone, water, and H_2_O_2_, to synthesize TiN NPs and clarify the effect of liquid type on the composition and properties of the formed NPs. The ablation in all solvents led to the formation of spherical NPs with a mean size depending on the liquid type, while the composition of the NPs ranged from partly oxidized TiN to almost pure TiO_2_, which conditioned variations of plasmonic peak in the region of relative tissue transparency (670–700 nm). The degree of NP oxidation depended on the solvent, with much stronger oxidation for NPs prepared in aqueous solutions (especially in H_2_O_2_), while the ablation in organic solvents resulted in a partial formation of titanium carbides as by-products. The obtained results contribute to better understanding of the processes in reactive PLAL and can be used to design TiN NPs with desired properties for biomedical applications.

## 1. Introduction

Titanium nitride (TiN) combines properties of covalent compounds, such as high mechanical and thermal stability, with metallic characteristics such as high thermal and electric conductivity [1]. TiN nanoparticles (NPs) have recently gained a huge boost of attention as an alternative plasmonic nanomaterial [2,3,4]. While the nanostructures of conventional plasmonic metals (silver, gold) exhibit their absorption/scattering resonance feature in ultraviolet (around 410 nm) and visible (around 520–550 nm), respectively, TiN NPs can provide a red-shifted plasmonic feature (around 670–700 nm) with a long tail over 800 nm [5,6], matching the window of relative tissue transparency (650–900 nm), which promises attractive biomedical applications [7,8]. Furthermore, TiN NPs predominantly dissipate absorbed electromagnetic energy into heat [6], which favors the use of these NPs in the photothermal therapy (PTT) of cancer. However, the synthesis of TiN NPs for biomedical applications is difficult within conventional chemical or plasma routes, as they are often contaminated by toxic byproducts, have a broad size and shape distribution, and are not easily water-dispersible. Indeed, chemical methods include reduction-nitridation reactions [9,10] employing hazardous reagents, which could cause toxicity issues. On the other hand, alternative physical methods [11,12] result in the production of TiN NPs in the form of dry powders. Therefore, similar wet chemistry steps are typically required to stabilize the colloidal solutions of these NPs.

Pulsed laser ablation in liquids (PLAL) is a low-cost and scalable method for the synthesis of stable colloidal solutions of bare (ligand-free) NPs [13,14]. In this method, a piece of solid material (a target) submerged into a liquid is irradiated by focused pulsed laser radiation. The laser-matter interaction results in “natural” production of NPs, which form colloidal NP solutions. Ultrashort laser ablation (femtosecond (fs) or picoseconds (ps)) has demonstrated the most efficient control of NPs among PLAL methods [14]. As an example, we used an fs laser-ablative technique to synthesize solutions of a variety of low size-dispersed nanomaterials, including Au [15,16], Si [17], and Bi [18] NPs. Recently, we adapted this technique for the synthesis of bare TiN NPs [19], which look extremely promising for biomedical applications due to high safety profiles both in vitro and in vivo [19,20]. We also demonstrated that the laser-synthesized TiN NPs are able to initiate the efficient photothermal destruction of cancer cells [19] and have a strong non-linear photoacoustic response, which can be used as an additional imaging channel [21].

It should be noted that PLAL is a very flexible technique, which can result in the formation of NPs with various chemical compositions from a single ablation target. This composition tuning can be achieved by ablation in liquids of different chemical origin. For example, PLAL of a metallic Cu target in water or H_2_O_2_ leads to the formation of Cu/CuO or Cu/CuO_2_ NPs, respectively [22]. As another example, the PLAL of metallic Ti target in different liquid ambient can lead to the formation of different compounds, including TiO_2_ [23], various titanium suboxides, TiO_x_ (0 < x < 2), titanium carbides, TiC [24], and oxycarbides, TiC_x_O_y_ [25]. In this context, one can expect similar compositional effects for TiN nanostructures under their laser-ablative synthesis in different liquid ambient, which could be used to obtain favorable surface chemistry and optical properties of formed NPs.

In this work, for the first time we systematically studied the effect of liquid ambient on the properties of TiN-based NPs synthesized by PLAL. To address the issue, we carried out experiments on the laser ablation of TiN target in five different liquid ambients, namely H_2_O_2_, water, acetone, dimethylformamide (DMF), and acetonitrile. We then systematically analyzed synthesized NPs using scanning electron microscopy (SEM), energy dispersive X-ray (EDX) spectroscopy, X-ray photoelectron spectroscopy (XPS), and spectrophotometry. Special attention was paid to the analysis of EDX data, for which we developed a statistical approach to account for variations of EDX signals from the NPs. The experimental data was carefully evaluated and discussed to clarify the effect of liquid ambient on the chemical composition and properties of the synthesized NPs. The presented results provide new insights into the processes that occur during reactive PLAL, and can be used to design TiN NPs with desired properties for various applications.

## 2. Materials and Methods

### 2.1. NPs Synthesis

To synthesize NPs, we used methods of pulsed laser ablation in liquids. A hot-pressed TiN target (1 × 20 × 20 mm plate, 99.5% purity, Girmet, Moscow, Russia) was fixed vertically in a 20 ml optical (ablation) cuvette. The target was irradiated horizontally by a laser beam from a Yb: KGW femtosecond laser (270 fs, 1030 nm, 30 uJ, 100 kHz, TETA-10, Avesta, Moscow, Russia) through a side wall of the ablation cuvette. The thickness of the liquid layer between the entrance glass of the cuvette and the surface of the ablation target was 3 mm. The laser beam was focused by a 100 mm working distance F-theta objective on the surface of the target. The lens–cuvette distance was 95–97 mm, while the exact value was adjusted independently for each liquid to ensure the highest productivity of the laser ablative synthesis. The laser beam was moved (scanned) along a spiral pattern on the target surface at the scanning velocity of 4 m/s using a galvanometric scanner. The duration of the ablation process was 10 min. The synthesis was performed in a series of liquids (Figure S1), namely: Acetone, DMF, acetonitrile, water, and hydrogen peroxide (H_2_O_2_). The obtained colloidal solutions were collected after the synthesis and examined without any additional post-processing.

### 2.2. NPs Characterization

Concentrations of NPs were determined by atomic absorption technique (AAS) using a MGA-1000 spectrometer (Lumex, Saint Petersburg, Russia), which provided information about the mass of Ti in the samples. Calibration of the spectrometer was performed using Ti standard calibration sample (Russian state standard sample #8464-2003). For measurements, colloidal solutions of NPs prepared by laser ablation were diluted 50,000-fold in 5% nitric acid. 20 uL of the final solution was measured three times, and the results were averaged. A complete temperature program for measurements is shown in Supplementary Materials Table S1.

Surface chemical states of the synthesized NPs were analyzed by X-ray photoelectron spectroscopy (XPS) using a Theta Probe tool (Thermo Scientific) under high-vacuum conditions using a monochromatic Al-Kα X-ray source (1486.6 eV). XPS spectra were measured using fixed analyzer transmission mode at 50 eV pass energy. The calibration was carried out by the C 1s peak centered at 284.5 eV.

Size, morphology, and semi-quantitative chemical composition of the synthesized NPs were measured by scanning electron microscopy (SEM) using an MAIA-3 electron microscope (Tescan, Brno, Czech Republic), equipped with energy dispersive X-ray (EDX) detector X-Act (Oxford Instruments, Abington, UK). SEM images were obtained at 30 keV accelerating voltage, while EDX spectra were collected at 20 keV. Samples for both SEM and EDX measurements were prepared by dropping 2 uL of colloidal solutions onto cleaned monocrystalline Si substrate, which was subsequently dried at ambient conditions.

Optical extinction spectra were measured using a PSI-MC 2 spectrophotometer (Sol Instruments, Minsk, Belarus) in glass cuvettes having 10 mm optical path length.

### 2.3. EDX Data Processing

To measure EDX spectra and obtain semi-quantitative information about the chemical composition of the synthesized NPs, we developed a deconvolution procedure, which included the following major steps, as shown in Figure S2. First, a background EDX spectrum obtained at a region free from NPs was subtracted from the experimental EDX spectrum obtained from a group of NPs (Figure S2A–C). Then, the background-corrected experimental spectrum was fitted with the Ti spectrum using Kα and Kβ lines of Ti, centered around 4.5 and 4.9 keV (Figure S2D). This procedure enabled us to scale the reference Ti spectrum to the experimental one. Subsequently, we subtracted L lines of the Ti spectrum (which span from 0.26 to 0.55 keV) from the experimental spectrum (Figure S2E). This was necessary, because the L lines of Ti largely overlap with the EDX spectra of C (spanning from 0.16 to 0.36 keV with the center at 0.27 keV), N (spanning from 0.27 to 0.5 keV with the center at 0.39 keV), and O (spanning from 0.36 to 0.64 keV with the center at 0.52 keV). Finally, the spectral region from 0.16 to 0.64 keV was fitted with C, N, and O spectra (Figure S2F). At least 10 different points were measured on each sample, and peak intensities were averaged for each chemical element.

A special sample preparation procedure was applied to measure the reference spectrum from the TiN target. The sample was prepared by mechanical grinding of the target into sub micrometer particles. The particles were then placed on a crystalline silicon wafer. The reference EDX spectrum was taken from particles, which had roughly the same volume as agglomerates of laser-synthesized TiN NPs, which were previously measured. This sample preparation procedure was applied to account for attenuation of the X-ray signal in the sample material, which could lead to significant underestimation of nitrogen content in bulk TiN samples.

## 3. Results and Discussion

### 3.1. Synthesis of Nanoparticles

We started our study by choosing a series of liquids (H_2_O_2_, water, DMF, acetone, acetonitrile), in which the TiN target was ablated. In this article, NPs synthesized in these liquids are referred to as H_2_O_2_-TiN, W-TiN, DMF-TiN, A-TiN, and AN-TiN NPs, respectively; however, it does not mean that composition of NPs was a stoichiometric TiN. The choice of liquids for the laser ablative synthesis was reasoned as follows. First, we used a pair of liquids, namely water and acetone, which were the most widely used for PLAL synthesis of TiN NPs in the literature [19,20,21]. Then, we used two nitrogen-containing liquids: one with an oxygen atom in its molecular composition (DMF) and the other without oxygen (acetonitrile). The rational for using these two liquids is based on the presence of nitrogen atoms in their chemical compositions, which could potentially preserve more nitrogen in the NPs composition and decrease their oxidation. This argument is supported by the fact that the alternative physical synthesis methods of TiN NPs include the treatment of titanium oxide particles in a nitrogen-rich atmosphere at elevated temperatures [26] and plasma-assisted routes in a nitrogen atmosphere [27]. Thus, at appropriate conditions, nitrogen could replace oxygen even in fully oxidized titanium NPs. At the same time, PLAL synthesis include stages (plasma stage and cavitation bubble stage), which contain products of solvent molecule decomposition [14] and involve high temperature and high pressure conditions. Therefore, we supposed that the use of nitrogen containing liquids in PLAL can provide conditions that are similar to conditions of alternative physical TiN NP synthesis routes. Thus, these liquids can potentially be favorable for higher yields of titanium nitrides, whereas the competing presence or absence of oxygen atoms in the liquid molecules (DMF or acetonitrile) could affect the oxidation process. We finally selected H_2_O_2_ as one of the most oxidizing liquids, which therefore should lead to the highest oxidation of the synthesized NPs for comparison reasons.

Laser ablation of the TiN target led to fast coloration of liquids in the ablation chamber, which indicated the formation of NPs. The color was dark blue, almost black, for all liquids except H_2_O_2_, for which the color was almost transparent with a light blue-green tint.

### 3.2. SEM Characterization

**Figure 1:**
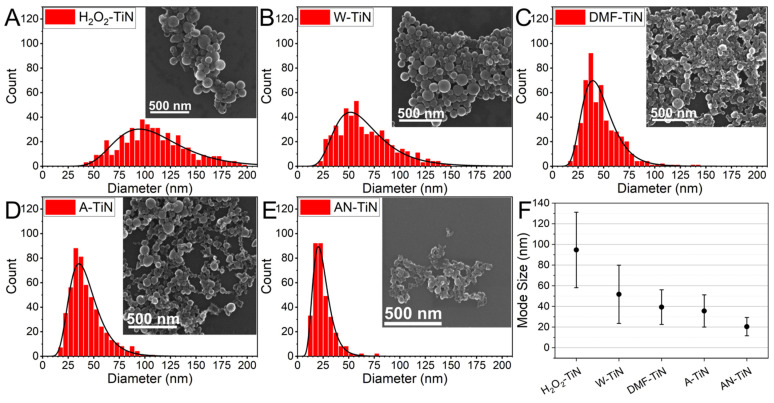
SEM characterization of NPs prepared by PLAL of TiN target in different liquids. (**A**–**E**) size histograms of H_2_O_2_-TiN, W-TiN, DMF-TiN, A-TiN, and AN-TiN NPs, respectively. Black curves are lognormal data fits. Insets demonstrate characteristic SEM images of the NPs. (**F**) Mode size of the lognormal fits of size distribution of NPs prepared in different liquids. Error bars represent distribution widths.

SEM imaging of the synthesized NPs revealed that the NPs had spherical morphology in all cases (Figure 1A–E). A significant number of H_2_O_2_-TiN NPs (up to 20–30%) had a morphology of ruptured shells, some characteristic images of which are shown in Figure S3. We already observed similar nanostructures in our previous studies [19,28] for NPs prepared by PLAL of TiN target in H_2_O_2_ and, to a lesser extent, in water; however, similar structures had never been observed in any organic solvents. A possible mechanism of the nano shell formation is discussed later in this article.

Statistical analysis of the SEM images showed that NP sizes were lognormally distributed, while the liquid ambient determined the mean size and the distribution width. Both values decreased as the liquids were replaced in the following sequence: H_2_O_2_–water–DMF–acetone–acetonitrile (Figure 1F and Table S2). The mean (modal) size of H_2_O_2_-TiN NPs was 95 nm, and some of the largest NPs were up to 200 nm, while the mean size of AN-TiN NPs was 20 nm, and almost no NPs exceeding 50 nm were observed. The tendency for the decrease of the NPs size in organic solvents could be explained by early capping of NPs formed just after the laser-target interaction, by solvent molecules, or by products of their decomposition similar to how it happens for other NPs synthesized by PLAL [14,29]. This explanation is indirectly supported by an amorphous carbon-containing matrix, which is often observed on SEM images of TiN NPs, synthesized in organic solvents, but not in water.

### 3.3. EDX Characterization

To study the chemical compositions of NPs, we developed a semi-quantitative statistical procedure to account for variations of EDX signals, which are often observed when NPs are measured. In this procedure, we first extracted data on the chemical elements of interest (titanium, nitrogen, oxygen, and carbon) and then statistically analyzed nitrogen and oxygen content normalized to titanium content in the NPs. The procedure details are described in the Materials and Methods section and in Figure S2.

**Figure 2:**
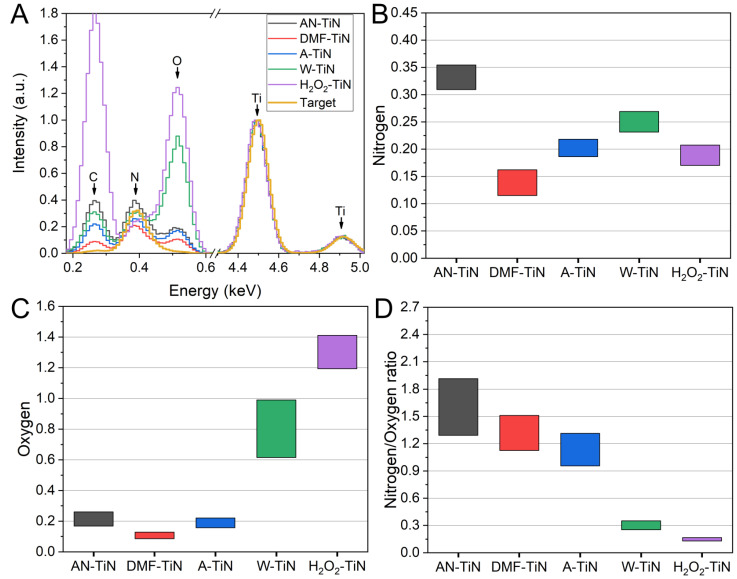
EDX characterization of synthesized TiN-based NPs. (**A**) Averaged background corrected EDX spectra (black line—AN-TiN NPs, red line—DMF-TiN NPs, blue line—A-TiN NPs, green line—W-TiN NPs, purple line—H_2_O_2_-TiN NPs, brown line—TiN ablation target). All spectra are normalized to titanium and averaged over all data points, measured on each sample. (**B**) Nitrogen and (**C**) oxygen content of synthesized NPs normalized to titanium. Values are calculated as the intensity of nitrogen peak at 0.39 keV or oxygen peak at 0.52 keV, divided by intensity of the Ti peak at 4.5 keV, respectively. (**D**) Ratio of nitrogen content to oxygen content in the NPs. Data in (**B**–**D**) are represented as mean ± standard deviation. At least seven different data points were measured on each sample.

Averaged EDX spectra of the NPs and an ablation target are shown in Figure 2A. As one can see, NPs synthesized in all liquids were oxidized, but the oxidation state was different. Indeed, AN-TiN, DMF-TiN, and A-TiN NPs were oxidized much weaker than H_2_O_2_-TiN and W-TiN NPs. Surprisingly, AN-TiN NPs had higher oxygen content than DMF-TiN NPs (and approximately equal oxygen content to A-TiN NPs), despite the absence of oxygen atoms in the molecular structure of acetonitrile. This result can be explained by a fast surface oxidation of the titanium nitride. Most of the TiN surface could be oxidized within several minutes, even at room temperature [30], while sample preparation procedure for the EDX measurements took several hours. Therefore, the surface of the analyzed NPs can be considered as almost fully covered by native oxides. Furthermore, AN-TiN NPs are almost two folds smaller than DMF-TiN or A-TiN NPs (Figure 1F), which means that AN-TiN NPs have a 4-fold large surface area. This could lead to overall higher oxygen signal from NPs prepared in acetonitrile due to the oxidation of a larger surface area. Another possible source of oxidation of AN-TiN NPs is atmospheric molecular oxygen dissolved in the acetonitrile, which could oxidize NPs during their formation by PLAL [29]. We tried to pump out the dissolved molecular oxygen O_2_ from the liquid by bubbling the liquids with N_2_ before and during the ablation process, but it did not lead to an increase of the nitrogen/oxygen ratio (data not shown).

Interestingly, the relative nitrogen signal from AN-TiN NPs was even higher than the nitrogen signal from the ablation target, which can be explained by the incorporation of nitrogen atoms from the decomposition of acetonitrile molecules into the NPs during the ablation process. However, the nitrogen content of DMF-TiN NPs was the lowest among all synthesized NPs, despite the presence of nitrogen in the molecular structure of DMF. Therefore, we suppose that the chemical state of nitrogen in the structure of liquid molecules appears to be an important factor for final composition of the laser-synthesized NPs. To further confirm this assumption, we performed PLAL of metallic Ti target in acetonitrile and DMF (AN-Ti NPs and DMF-Ti NPs, accordingly). The analysis of EDX spectra of the synthesized NPs (Figure S4) showed that AN-Ti NPs had several times higher nitrogen content than DMF-Ti NPs, which supports our assumption.

Figure 2B–D demonstrates statistical EDX information about nitrogen and oxygen and their ratio in NPs synthesized in different liquid ambients. As one can see, the main difference was in the amount of oxygen in the NP composition, while nitrogen signal level was more stable. NPs prepared in acetonitrile, DMF, and acetone had high (1.1–1.6) mean values of nitrogen/oxygen ratio with the highest value for AN-TiN NPs. At the same time, W-TiN and H_2_O_2_-TiN NPs had very low (0.15–0.3) nitrogen/oxygen values, which suggests that not only the surface, but also the core of the NPs was oxidized.

### 3.4. XPS Characterization

**Figure 3:**
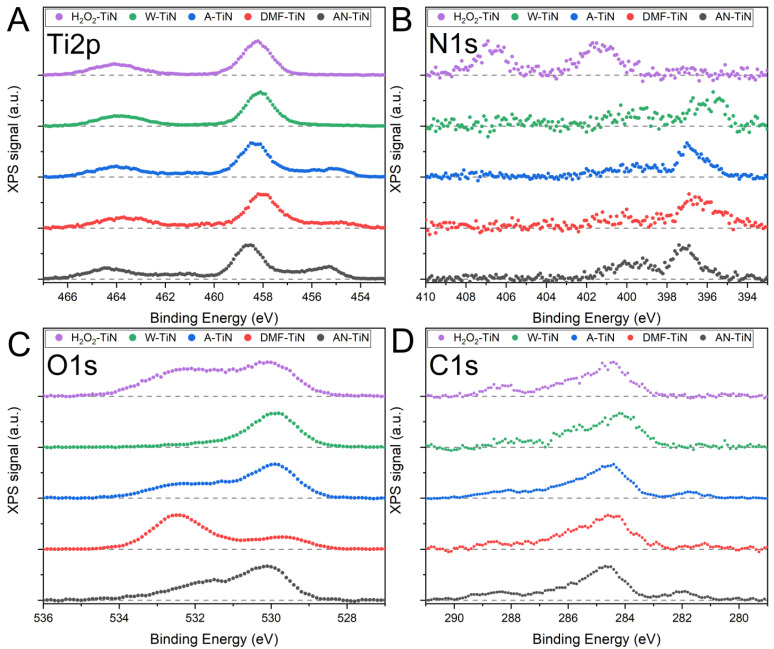
XPS characterization of the synthesized NPs. (**A**) Ti 2p, (**B**) N 1s, (**C**) O 1s, and (**D**) C 1s electronic levels of H_2_O_2_-TiN (purple), W-TiN (green), A-TiN (blue), DMF-TiN (red), and AN-TiN (black) NPs.

The surface chemistry of the synthesized NPs was studied by XPS. We measured binding energies of Ti 2p, N 1s, O 1s, and C 1s electrons. The obtained results are summarized in Figure 3 and in Table 1 and Table 2. A detailed deconvolution of XPS spectra is presented in Figure S5.

**Table 1:** Results of deconvolution of Ti 2p and N 1s XPS spectra of the synthesized NPs. Values in parenthesis represent alternative six-peak fit for W-TiN sample.

Ti 2p						
Bond	Sample	Peak Position, eV		Relative Peak Intensity, %		Reference
		2p_3/2_	2p_1/2_	2p_3/2_	2p_1/2_	
TiN	AN-TiN	455.4	461	34	15	[27,31,32,33,34,35]
	DMF-TiN	454.9	460.3	16	13	
	A-TiN	455.2	460.8	25	14	
	W-TiN	(454.9)	(459.8)	(3)	(7)	
	H_2_O_2_-TiN	-	-	-	-	
TiN, TiNO	AN-TiN	457.1	463.3	20	8	[31,32,33,34,36]
	DMF-TiN	456.8	462.8	22	12	
	A-TiN	457.1	463.1	19	9	
	W-TiN	(456.9)	(462.7)	(9)	(6)	
	H_2_O_2_-TiN	-	-	-	-	
TiO_2_	AN-TiN	458.6	464.4	100	26	[33,37]
	DMF-TiN	458.1	464	100	26	
	A-TiN	458.4	464.2	100	27	
	W-TiN	458.2	464	100	29	
	H_2_O_2_-TiN	458.2	464.1	100	31	
**N 1s**						
**Bond**	**Sample**	**Peak position, eV**		**Relative peak intensity, %**		**Reference**
TiN	AN-TiN	397.1		100		[31,32,33,35,36]
	DMF-TiN	396.4		100		
	A-TiN	396.7		100		
	W-TiN	395.9		100		
	H_2_O_2_-TiN	-		-		
TiNO	AN-TiN	399.8		53		[31,32,33,35,36]
	DMF-TiN	399.6		30		
	A-TiN	399.7		35		
	W-TiN	399.6		47		
	H_2_O_2_-TiN	-		-		
N_2_, NO_x_	AN-TiN	-		-		[33,38,39,40]
	DMF-TiN	-		-		
	A-TiN	-		-		
	W-TiN	-		-		
	H_2_O_2_-TiN	401.4	406.7	100	90	

The Ti 2p spectra of all samples (Figure 3) are dominated by a doublet of symmetrical peaks located at 458.3 ± 0.3 and 464.2 ± 0.2 eV. The positions of these peaks agree with literature values of Ti 2p_3/2_ and Ti 2p_1/2_ peaks of TiO_2_ [33,37]. For crystalline (anatase, rutile) TiO_2_, the intensity ratio of these peaks should be close to 100:50, while for amorphous titanium oxide it increases [33]. The values of this ratio for the synthesized NPs are between 100:26 and 100:31. Therefore, the analysis of XPS data suggests that the surface of NPs consists of predominantly amorphous TiO_2_. Note that the storage of H_2_O_2_-TiN NPs for several months leads to a reduction of the intensity ratio to almost 100:50 (Figure S6), which suggests that the surface TiO_2_ becomes more crystalline under aging.

Further deconvolution (Table 1, Figure S5) of the Ti 2p XPS spectra indicates the presence of two more doublets for AN-TiN, DMF-TiN, and A-TiN samples. Note that the Ti 2p XPS spectra of W-TiN, and especially H_2_O_2_-TiN, can be reasonably well fitted just by two peaks of TiO_2_ (Figure S5D,E first panel); however, the deconvolution of W-TiN NPs XPS spectra into six peaks gives a better fit (Figure S7). A very low (comparable to noise) intensity of TiN peaks confirms that the surfaces of W-TiN and H_2_O_2_-TiN NPs are almost completely oxidized. The first doublet at 455.1 ± 0.3 and 460.6 ± 0.8 eV is generally attributed to Ti 2p_3/2_ and Ti 2p_1/2_ of the Ti-N bond [27,33,34,35]. Note that the position of the TiN Ti 2p_3/2_ (and Ti 2p_1/2_ accordingly) varies for different samples (455.4 eV for AN-TiN, 454.9 eV for DMF-TiN, and 455.2 eV for A-TiN). Several reasons could explain these shifts. One of reasons can be related to the oxidation of the NP surface. Indeed, the influence of the native oxide on the position of TiN 2p_3/2_ peak was already mentioned in the literature [32,34]. As another reason, the shifts in the position of the TiN 2p_3/2_ could be due to the sub-stoichiometric nature of TiN in the synthesized NPs. Indeed, Porte et al. [34] studied sub-stoichiometric TiN_x_ (0.5 < x < 1) and found that the position of the peak can shift by up to ~0.6 eV under the change of stoichiometry from TiN_0.5_ to TiN. At the same time, it is very likely that NPs produced by PLAL of the TiN target can have a sub-stoichiometric nature, and this could be a reason for the shifts of the TiN 2p_3/2_ peak position.

The value of intensity ratio of Ti 2p_3/2_ peaks of TiN (455.1 eV) and TiO_2_ (458.3 eV) can be used as a qualitative measure of relative abundance of surface titanium nitrides and oxides. The increase of this value means the increase in abundance of surface titanium nitrides over oxides. This intensity ratio increases 3% for W-TiN NPs (unreliable fit) to 34% for AN-TiN NPs, while no TiN peak can be found for H_2_O_2_-TiN NPs at all. This data suggests that AN-TiN NPs have the highest relative abundance of TiN bonds near the surface. Note that this doublet could also be attributed to the formation of TiC [41]. The last doublet of intermediate peaks centered at 457.0 ± 0.2 and 463 ± 0.3 eV is the most controversial. Some authors attribute them to titanium oxynitrides [33,36], while it was also demonstrated that these peaks could indicate sub-stochiometric titanium nitride [34].

The analysis of N 1s XPS spectra (Figure 3B and Table 1) demonstrate significant differences in XPS spectra between H_2_O_2_-TiN NPs and other types of NPs. AN-TiN, DMF-TiN, A-TiN, and W-TiN NPs demonstrate strong peak at 396.5 ± 0.6 eV and a smaller peak at 399.7 ± 0.1 eV (Figure S5). These peaks should be attributed to Ti-N bond and titanium oxynitride accordingly [33,35,36]. Note that the W-TiN NPs sample has very low intensity of the N 1s signal, while the position of its main peak is shifted to lower binding energies (395.9 eV). We believe that this can be attributed to TiNO rather than TiN [42]. The intensity ratio of Ti-N bond in N 1s level to TiO_2_ bond in Ti 2p_3/2_ level (11.2% for AN-TiN, 8.3% for DMF-TiN, 7.3% for A-TiN, and 1.7% for W-TiN) also demonstrates that AN-TiN NPs have the highest relative amount of surface nitrogen, while W-TiN NPs have the lowest amount. H_2_O_2_-TiN NPs have a completely different N 1s XPS spectrum. These NPs have two equally intense peaks at 401.4 and 406.7 eV. A possible origin of these peaks is adsorbed molecular N_2_ [33,38,39]. Another possible origin of these peaks is the formation of NO_x_ bonds [40]. In any case, no Ti-N chemical bonds can be found for H_2_O_2_-TiN NPs.

The O 1s XPS spectra of the synthesized NPs can be fitted by three peaks (Figure 3C and Figure S5) centered at 529.8 ± 0.3, 531.4 ± 0.3, and 532.4 ± 0.2 eV, with the only exception of W-TiN NPs, in which O 1s XPS spectrum can be fitted by only two peaks (529.9 and 531.2 eV). The peak at 529.8 eV should be attributed to titanium oxide [33,36]. The origin of the peak at 531.4 is very controversial; it can be attributed to oxynitride [36] or OH^-^ [37,39,43]. The peak at 533.4 should be attributed to H_2_O [39], which could appear from incomplete drying of samples.

We also analyzed C 1s XPS spectra because EDS data indicated the presence of carbon in the samples. Note that the carbon signal does not necessarily come from NPs but could originate from impurities during the sample preparation procedure (drying of liquid droplets) of from common contamination of vacuum chambers by organics. The C 1s XPS spectra of all synthesized NPs were mostly similar. The most prominent peaks were centered at 284.4 ± 0.3, 285.9 ± 0.3, and 288.3 ± 0.4 eV, which belong to C-C, C-O, and C=O bonds, respectively [44]. However, the C 1s XPS spectra of AN-TiN, DMF-TiN, and A-TiN NPs contain a peak centered at 281.7 ± 0.4 eV, which is absent in the spectra of W-TiN and H_2_O_2_-TiN NPs. The peak at 281.7 eV is generally attributed to inorganic carbides and therefore can be ascribed to the Ti-C bond [41]. The intensity ratio of N 1s peak at 396.5 eV (Ti-N bond) to the C 1s peak at 281.7 eV (Ti-C bond) is 1.36 for AN-TiN NPs, 1.41 for A-TiN NPs, and 2.28 for DMF-TiN NPs, which means that a significant amount of titanium carbides is formed (at least near the NPs surface) during PLAL in organic solvents.

The overall XPS analysis suggests that the surface of H_2_O_2_-TiN and W-TiN NPs consists primarily of amorphous TiO_2_ with an insignificant amount of Ti-N bonds for W-TiN NPs, while the surface of AN-TiN, DMF-TiN, and A-TiN NPs consist of titanium oxides, nitrides, carbides, and possibly oxynitrides.

**Table 2:** Results of deconvolution of O 1s and C 1s XPS spectra of the synthesized NPs.

**O 1s**				
**Bond**	**Sample**	**Peak Position, eV**	**Relative Peak Intensity, %**	**Reference**
TiO_2_	AN-TiN	530.1	100	[33,36]
	DMF-TiN	529.6	36	
	A-TiN	529.9	100	
	W-TiN	529.9	100	
	H_2_O_2_-TiN	530	100	
NO, OH^−^	AN-TiN	531.6	58	[33,37,39,43]
	DMF-TiN	531.6	29	
	A-TiN	531.2	21	
	W-TiN	531.2	19	
	H_2_O_2_-TiN	531.2	11	
H_2_O	AN-TiN	533.2	11	[33,37]
	DMF-TiN	532.6	100	
	A-TiN	532.3	43	
	W-TiN	-		
	H_2_O_2_-TiN	532.2	86	
**C 1s**				
TiC	AN-TiN	282	25	[41]
	DMF-TiN	281.2	13	
	A-TiN	281.7	23	
	W-TiN	-	-	
	H_2_O_2_-TiN	-	-	
CC	AN-TiN	284.7	100	[44]
	DMF-TiN	284.5	100	
	A-TiN	284.5	100	
	W-TiN	284.2	100	
	H_2_O_2_-TiN	284.5	100	
CO	AN-TiN	286.2	36	[44]
	DMF-TiN	286.1	46	
	A-TiN	285.7	60	
	W-TiN	285.8	53	
	H_2_O_2_-TiN	286.1	45	
C=O	AN-TiN	288.5	25	
	DMF-TiN	288.6	21	
	A-TiN	288.3	26	[44]
	W-TiN	287.9	21	
	H_2_O_2_-TiN	288.4	34	

### 3.5. Optical Characterization

**Figure 4:**
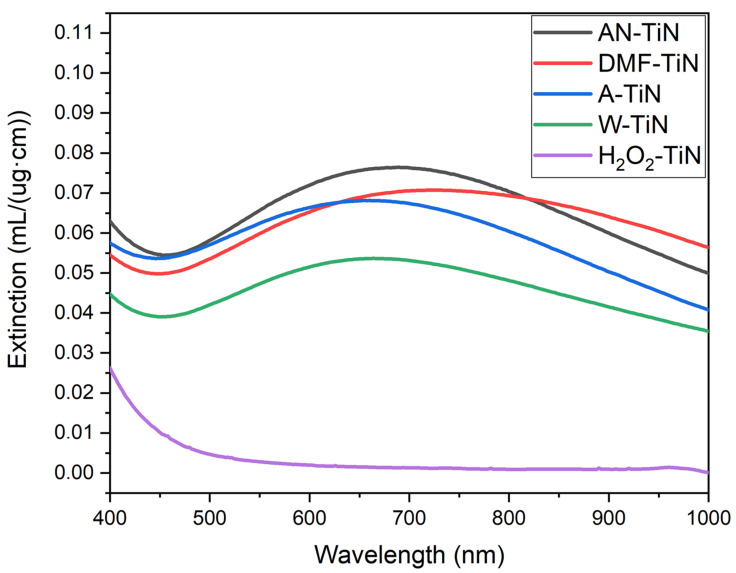
Optical extinction spectra of H_2_O_2_-TiN (purple), W-TiN (green), A-TiN (blue), DMF-TiN (red), and AN-TiN (black) NPs measured in 10 mm optical cuvettes and normalized to mass concentration of Ti in ug/mL.

We finally measured the optical extinction spectra of TiN-based NPs in the visible and near-IR range. The spectra, normalized to mass concentration of Ti, are shown in Figure 4. As one can see, NPs synthesized in organic solvents (AN-TiN, DMF-TiN, A-TiN) exhibit broad peaks centered between 650 and 750 nm, with similar values of optical extinction in the near-IR. NPs ablated in nitrogen-containing liquids (AN-TiN and DMF-TiN) have slightly higher (~10%) optical extinction in near-IR than A-TiN NPs. W-TiN NPs have approximately 20% smaller value of the optical extinction due to the higher content of titanium oxide in the NP composition. H_2_O_2_-TiN NPs almost do not interact with light in red and near-IR range. This behavior is typical for TiO_2_ NPs [45], which additionally confirms that H_2_O_2_-TiN NPs are almost completely oxidized.

### 3.6. Discussion

Our data demonstrate the influence of a liquid ambient on the composition and properties of TiN NPs synthesized by PLAL. The results agree with previously reported data on laser-ablative synthesis using reactive chemical species [22,23,24,25].

The strongest effect is achieved in H_2_O_2_. In this case, Ti 2p XPS and optical characterization suggests the formation of almost pure TiO_2_. The results are also supported by EDX data, which indicate very high oxygen content in the H_2_O_2_-TiN NPs. However, EDX and N 1s XPS data also indicate the presence of nitrogen in the H_2_O_2_-TiN NPs. The analysis of the N 1s XPS spectrum suggests that the origin of this signal is not related to any kind of Ti-N bonds but can be attributed to adsorbed molecular nitrogen N_2_ or nitrogen oxides. At the same time, SEM imaging evidences the formation of characteristic voids inside H_2_O_2_-TiN NPs, which lead to the formation of ruptured shells-like NPs (Figure S3). These two features (XPS spectra of adsorbed molecular N_2_ or NO_x_ and ruptured shells-like shape) were uniquely found in H_2_O_2_-TiN NPs during our study. Therefore, we believe that these features are linked and the voids could be filled with N_2_ or nitrogen oxide gas. We suppose that the gas originates from the TiN sample during formation of the NPs in highly oxidizing medium (H_2_O_2_). In the ruptured shell-like nanostructures, a part of this nitrogen-containing gas remains adsorbed at the inner surface of the NPs. In this case, XPS can detect peaks at 401.4 and 406.7 eV, which correspond to the presence of adsorbed gas molecules near the edges of the ruptured shells. These gas molecules (both adsorbed and inside voids) lead to the appearance of a nitrogen signal in the EDX spectra of H_2_O_2_-TiN NPs (Figure 2), but they do not affect the optical spectrum of the H_2_O_2_-TiN NPs. Therefore, despite nitrogen being present inside the H_2_O_2_-TiN NPs, the optical spectrum of the NPs corresponds to the spectrum of TiO_2_ NPs (Figure 4). Although this assumption seems logical and indirectly supported by all the experimental techniques used in this study, it still requires further direct confirmation.

The use of organic solvents (acetone, acetonitrile, DMF) in PLAL yields similar results. The NPs have close ratios of nitrogen to oxygen content, exhibit TiN, TiC, and possibly TiNO bonds on their surface. Oxygen in these NPs is present in the form of amorphous TiO_2_ and oxynitrides, while most of it originates from the fast surface oxidation of the NPs. The use of nitrogen-containing liquids for the preservation of nitrogen in the NPs composition turned out to be not straightforward. AN-TiN NPs demonstrate the highest value of nitrogen to oxygen ratio in the NPs composition (even higher nitrogen content than in the TiN target) and the strongest XPS peaks of TiN bonds. However, properties of DMF-TiN NPs are almost indistinguishable from those of A-TiN NPs. Therefore, we can conclude that not only the presence of a chemical element in the composition of a liquid molecule, but also the chemical state of this element, plays an important role in the formation of NPs by PLAL. Note that some titanium carbides can also be formed during ablation of TiN in organic solvents. Indeed, the intensity of Ti-C peak in C 1s XPS spectra is of the same order (although 1.4–2.2 fold lower), as the intensity of the Ti-N peak in N 1s XPS spectra of the same samples. At the same time, both Ti-N and Ti-C bonds appear at the same binding energy in Ti 2p XPS, and therefore Ti 2p XPS data cannot be used for the discrimination of these bonds. Furthermore, optical spectra in the visible and near-IR region are very similar for both titanium nitrides [6] and titanium carbides [46]. Moreover, the formation of titanium carbides was previously reported for PLAL of Ti target in organic solvents [24].

W-TiN NPs have intermediate properties between H_2_O_2_-TiN on one side and AN-TiN, DMF-TiN, and A-TiN on the other. The formation of various titanium oxides has already been described in the literature for the PLAL of Ti target in water [23,24]. The oxidation degree was found to decrease while the duration of laser pulses decreases from ms [23] to ns [24]. Our data indicate that even fs PLAL cannot exclude oxidation of Ti in water. We found that the surface of W-TiN NPs is almost fully oxidized without any sign of TiC on it. EDX data also suggest a high degree of NP oxidation, but optical spectra indicate the conservation of a significant TiN fraction. This experimental fact can be explained by a predominant oxidation of the NP surface, while the core remains unaffected. Similar oxidation behavior was reported for PLAL of Ti in water [24].

The synthesized TiN NPs present very appealing objects for biomedical and energy applications due to their high and wide optical absorption in near-IR spectral range. From this point of view, AN-TiN, DMF-TiN, and A-TiN seem to be especially relevant, as their optical mass extinction coefficients in the near-IR region (0.06–0.075 mL/(ug·cm)) are higher than values reported in the literature for NPs, which are tested for PTT [47,48,49,50]. In particular, the mass extinction coefficient at 808 nm of Au nanorods is 0.014 mL/(ug·cm) [47], nanosized graphene oxide is 0.025 mL/(ug·cm) [48], WS_2_ NPs is 0.024 mL/(ug·cm) [49], and MoS_2_ NPs is 0.028 mL/(ug·cm) [50].

The size of NPs is another important parameter, especially for biomedical applications. We established a strong dependence of the NPs size on the type of the solvent. Indeed, the mean size can be tuned from 20 to 100 nm (Figure 1F) by a simple change of the liquid ambient. Here, being the smallest (20 nm) among all the synthesized NPs, AN-TiN NPs look to be the best candidates for biomedical applications. However, the formation of toxic hydrogen cyanide during PLAL in acetonitrile could present a substantial difficulty in their biomedical applications, therefore further safety evaluation of AN-TiN NPs is required. W-TiN NPs can be treated as a good compromise for practical applications due to the simplicity of their preparation and the absence of any organic contamination, although they exhibit ~20% smaller optical extinction coefficient and have a slightly larger size (50 nm).

## 4. Conclusions

We presented a systematic study of NPs prepared by PLAL of a TiN target in different solvents, including acetonitrile, DMF, acetone, water, and H_2_O_2_. We showed that in all cases spherical NPs are formed. We also demonstrated that the size of the formed NPs depends on a liquid ambient type. AN-TiN and H_2_O_2_-TiN NPs have the smallest (20 nm) and largest (90 nm) mean sizes, respectively, while the sizes of other NPs were within the 40–50 nm range. All synthesized NPs had a high-oxidation state compared to the target material. The surface of W-TiN and H_2_O_2_-TiN NPs was almost fully oxidized. We also found that AN-TiN, DMF-TiN, and A-TiN NPs contained a slight titanium carbide fraction, which probably appeared due to solvent decomposition. AN-TiN, DMF-TiN, and A-TiN NPs demonstrated strong and wide optical extinction feature in the near-IR range (centered between 600 and 900 nm) with very high mass extinction coefficient of 0.06–0.075 mL/(ug·cm), which is very important for projected biomedical applications. The optical extinction of W-TiN NPs was 20% lower in the near-IR compared to NPs synthesized in organic solvents, but still significant, while H_2_O_2_-TiN did not exhibit any extinction in this range.

## Data Availability

Data is available on reasonable request from the corresponding author.

## Supplementary Materials

The following supporting information can be downloaded at: https://www.mdpi.com/article/10.3390/nano12101672/s1, Figure S1. List of liquids, which were used as mediums for the laser ablative synthesis; Figure S2. EDX deconvolution procedure. (A) A typical SEM image with an experimental and a background data point marked by red circles. (B) An experimental (black line) and a background (red line) EDX spectra obtained at data points marked in (A). (C) A EDX spectrum obtained by subtraction of the background spectrum from the experimental spectrum. (D) Fit of the EDX spectrum from (C) (black line) by an “ideal” Titanium (red line) EDX spectrum. (E) A EDX spectrum obtained by subtraction of the titanium spectrum from the experimental spectrum. (F) Final fit of the experimental EDX spectrum by EDX spectra of carbon (C, red line), nitrogen (N, blue line), and oxygen (O, green line); Figure S3. SEM images of NPs synthesized by PLAL in H2O2. Red circles highlight raptured NPs; Figure S4. Averaged background corrected EDX spectra of NPs, synthesized by PLAL of Ti target in DMF (DMF-Ti, black line) and acetonitrile (AN-TiN, red line); Figure S5. Deconvolution of XPS spectra of the synthesized NPs. (A) AN-TiN NPs; (B) DMF-TiN NPs; (C) A-TiN NPs; (D) W-TiN NPs; (E) H2O2-TiN NPs; Figure S6. Ti 2p XPS spectra of H2O2-TiN NPs after storage for several months; Figure S7. Alternative six-peak fit of XPS spectra of Ti 2p level of W-TiN NPs sample; Table S1. Temperature program for measurement mass of Ti in TiN NPs using AAS technique; Table S2. Statistical parameters of lognormal fits of sizes of NPs, synthesized in different liquids. μ and σ are parameters of lognormal distributions.
