# Supplementary material for: Synthesis of Titanium Nitride Nanoparticles by Pulsed Laser Ablation in Different Aqueous and Organic Solutions
Source: Nanomaterials (Basel). 2022 May 13;12(10):1672. doi: 10.3390/nano12101672 (PMC9147655; doi:10.3390/nano12101672)
Supplement: Supplementary file 1 [file nanomaterials-12-01672-s001.zip › nanomaterials-1730864-supplementary.pdf]

Supplementary Materials

Synthesis of Titanium Nitride Nanoparticles by Pulsed Laser Ablation in Different Aqueous and Organic Solutions

Anton A. Popov [1,*], Gleb V. Tikhonowski [1], Pavel V. Shakhov [1], Elena A. Popova-Kuznetsova [1], Gleb I. Tselikov [2], Roman I. Romanov [1], Andrey M. Markeev [2], Sergey M. Klimentov [1] and Andrei V. Kabashin [3,*]

1 National Research Nuclear University MEPhI, 115409 Moscow, Russia; gtikhonowski@gmail.com (G.V.T.); pvshakhov@mephi.ru (P.V.S.); eapopovakuznetsova@mephi.ru (E.A.P.-K.); limpo2003@mail.ru (R.I.R.); smklimentov@mephi.ru (S.M.K.)
2 Center for Photonics and 2D Materials, Moscow Institute of Physics and Technology, 141700 Dolgoprudny, Russia; celikov@physics.msu.ru (G.I.T.); markeev.am@mipt.ru (A.M.M.)
3 Laboratory LP3, Campus de Luminy, Aix-Marseille University, CNRS, 13288 Marseille, France
* Correspondence: aapopov1@mephi.ru (A.A.P.); andrei.kabashin@univ-amu.fr (A.V.K.)

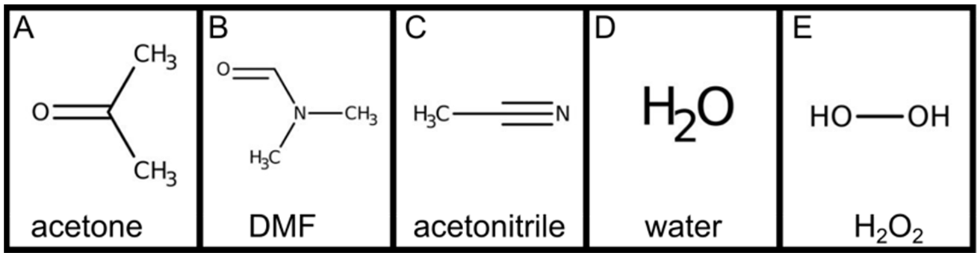

Figure S1. List of liquids, which were used as mediums for the laser ablative synthesis.

Table S1. Temperature program for measurement mass of Ti in TiN NPs using AAS technique.

Stage	Temperature, °C	Duration, s	Argon flow
Drying	120	30	Low
Pyrolysis 1	750	30	Low
Pyrolysis 2	1700	5	Low
Background estimate	750	6	Off
Atomization	2700	2	Off
Cleaning	2750	2	Low

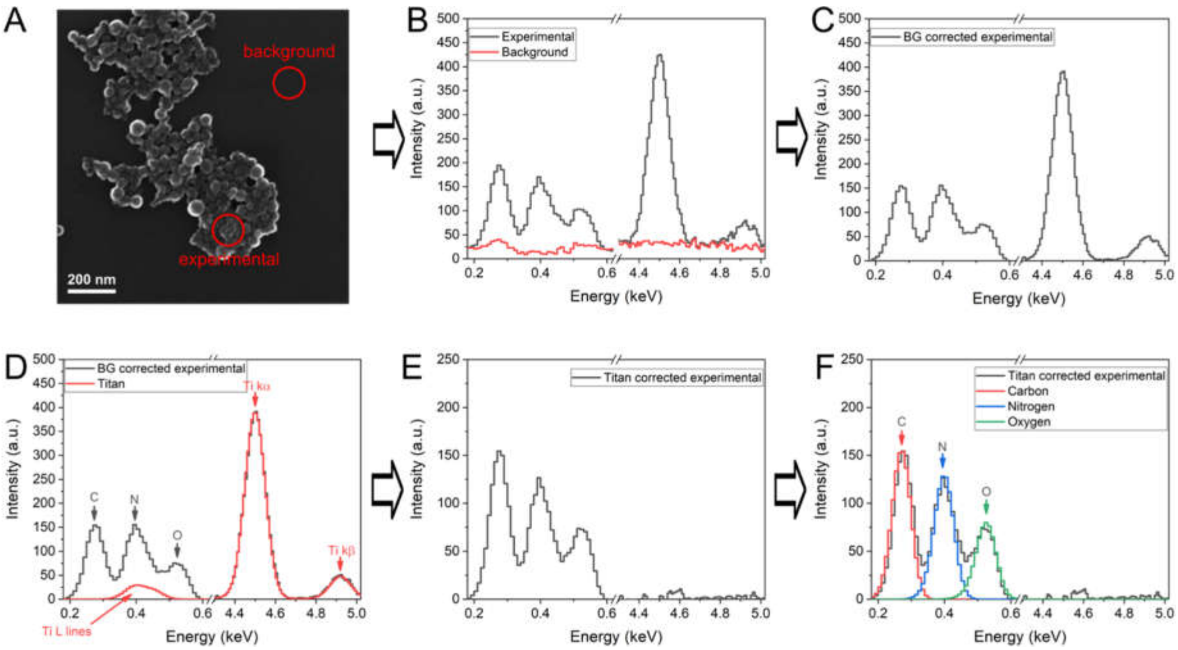

Figure S2. EDX deconvolution procedure. (A) Typical SEM image with an experimental and a background data points marked by red circles. (B) Experimental (black line) and background (red line) EDX spectra obtained at data points from marked in (A). (C) A EDX spectrum obtained by subtraction of the background spectrum from the experimental spectrum. (D) Fit of the EDX spectrum from (C) (black line) by an "ideal" Titanium (red line) EDX spectrum. (E) A EDX spectrum obtained by subtraction of the titanium spectrum from the experimental spectrum. (F) Final fit of the experimental EDX spectrum by EDX spectra of carbon (C, red line), nitrogen (N, blue line) and oxygen (O, green line).

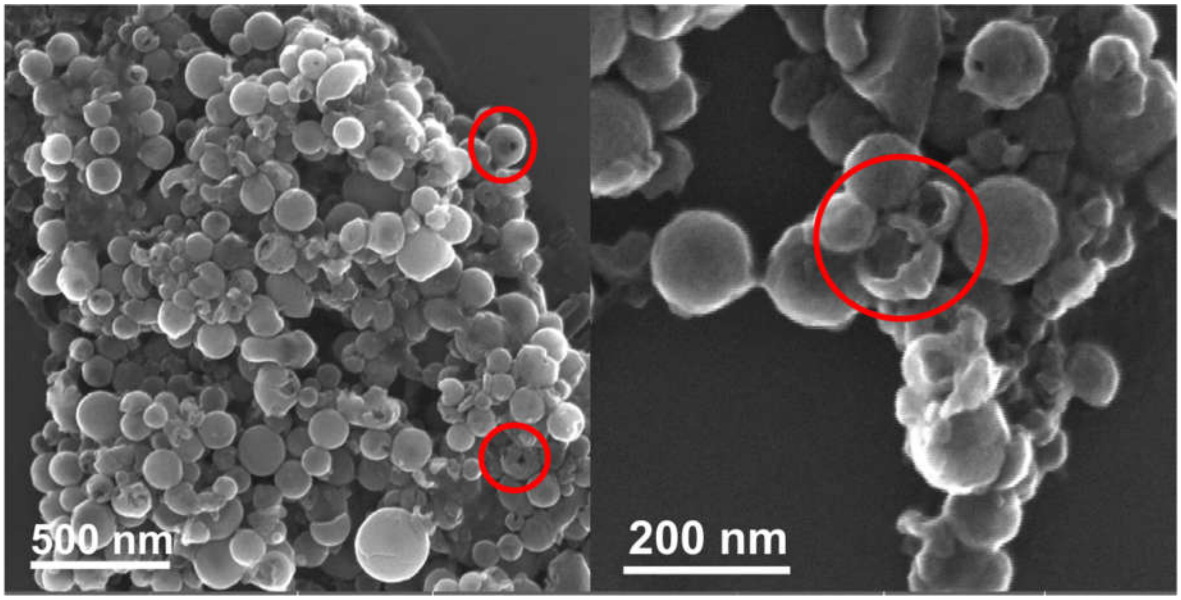

Figure S3. SEM images of NPs, synthesized by PLAL in H_2O_2. Red circles highlight raptured NPs.

Table S2. Statistical parameters of lognormal fits of sizes of NPs, synthesized in different liquids.
μ and σ are parameters of lognormal distributions.

Liquid	Mode, nm	Mean, nm	Variance, nm^2	μ	σ
H_2O_2	95	111	1336	4.654	0.322
Water	52	66	795	4.111	0.408
DMF	39	47	282	3.792	0.346
Acetone	36	43	244	3.697	0.353
Acetonitrile	20	25	78	3.138	0.350

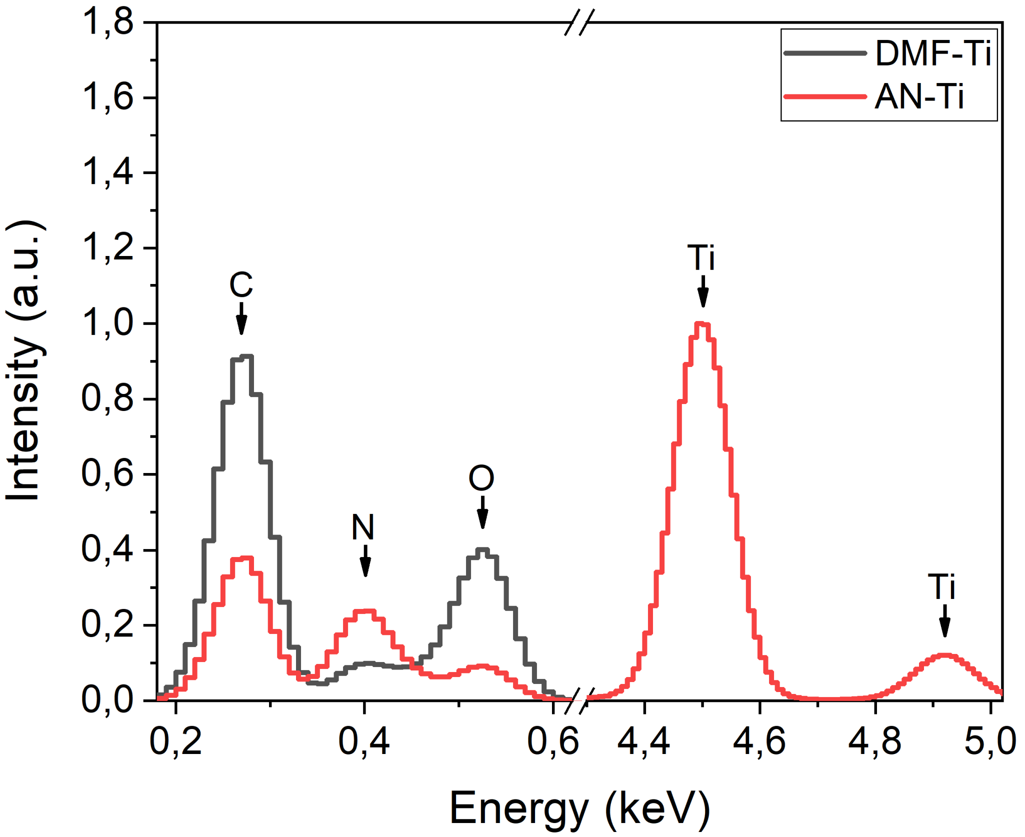

Figure S4. Averaged background corrected EDX spectra of NPs, synthesized by PLAL of Ti target in DMF (DMF-Ti, black line) and acetonitrile (AN-TiN, red line).

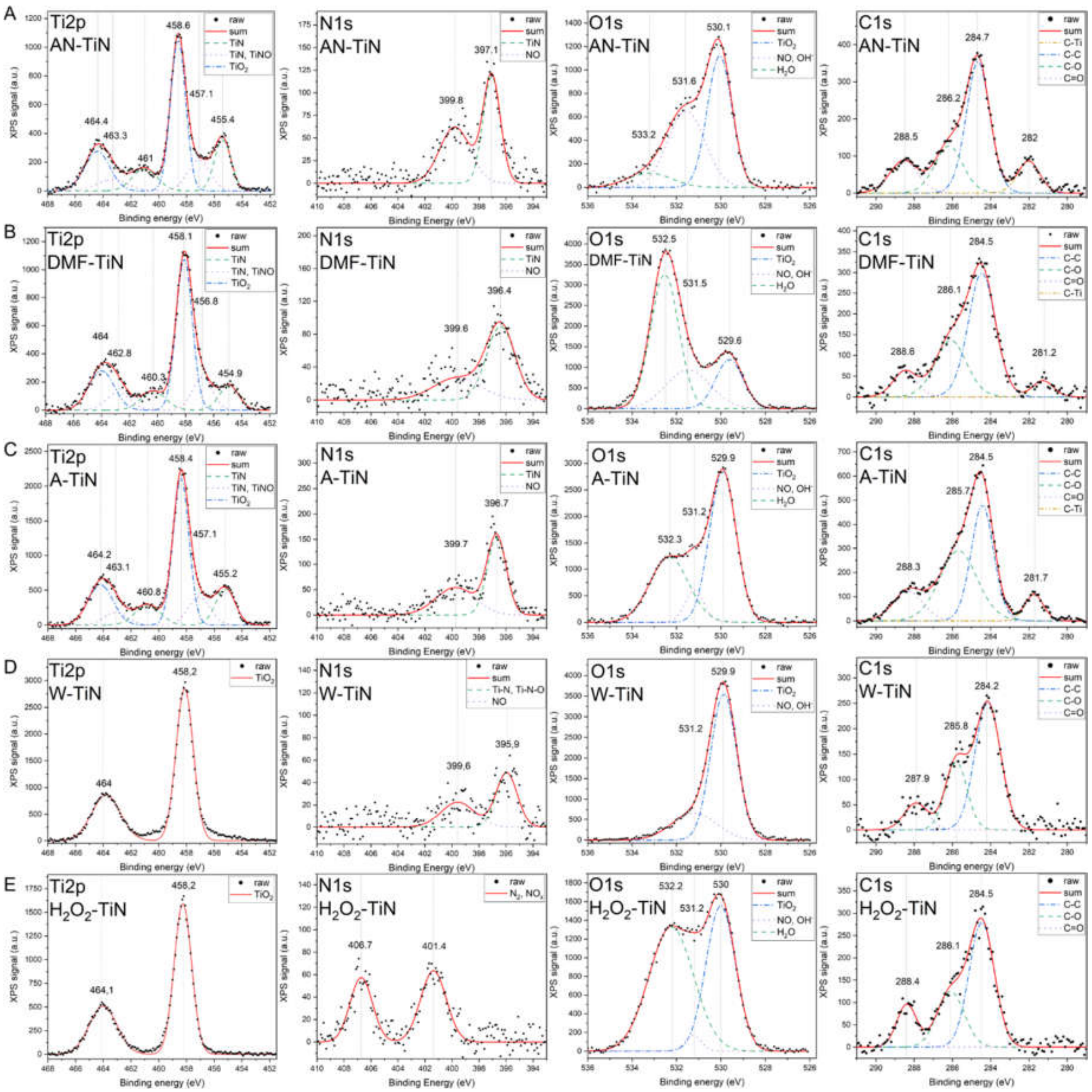

Figure S5. Deconvolution of XPS spectra of the synthesized NPs. (A) AN-TiN NPs; (B) DMF-TiN NPs; (C) A-TiN NPs; (D) W-TiN NPs; (E) H_2O_2-TiN NPs.

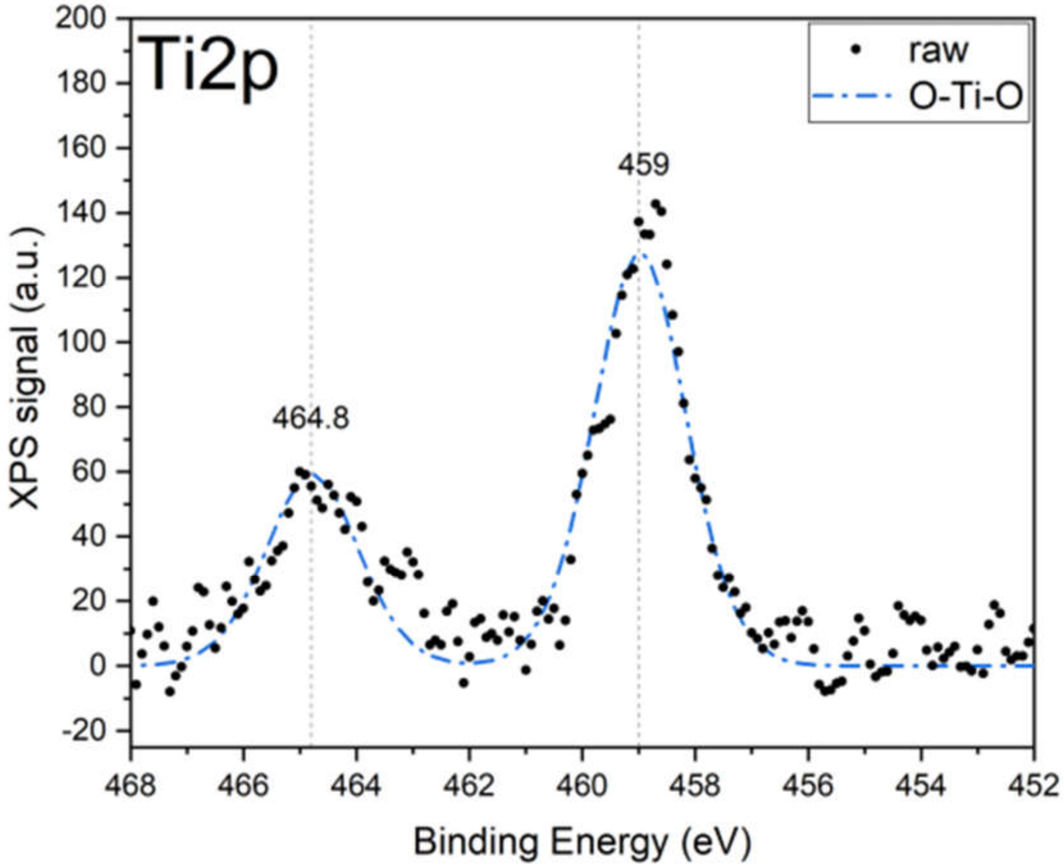

Figure S6. Ti 2p XPS spectra of H2O2-TiN NPs after storage for several months.

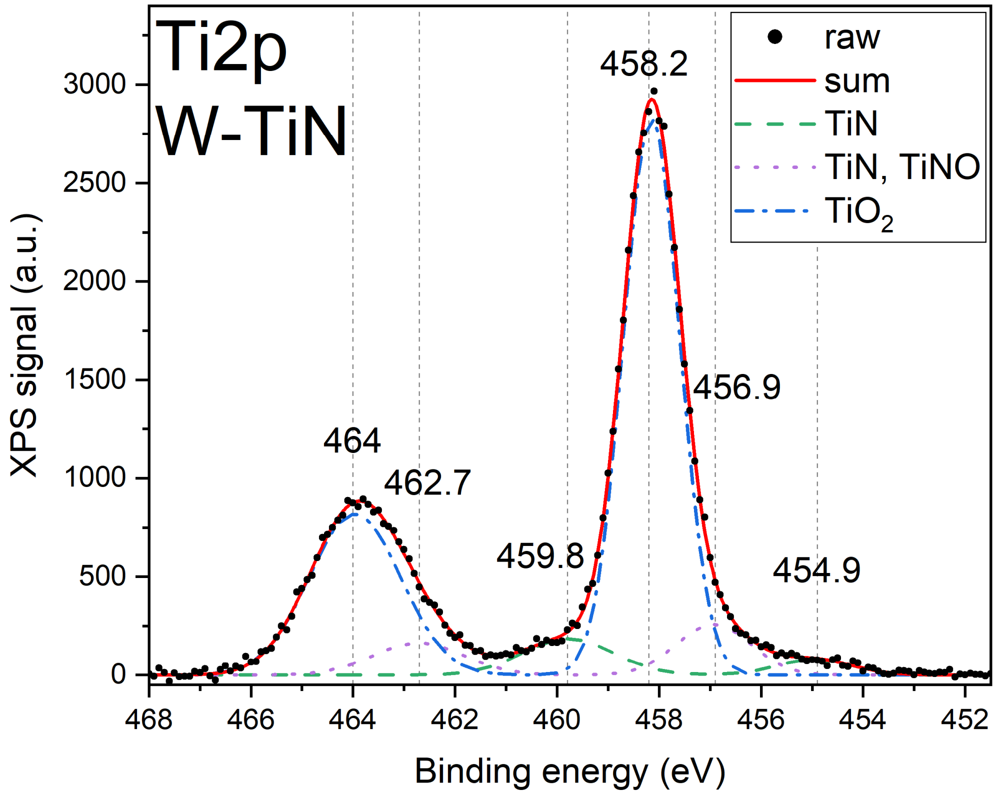

Figure S7. Alternative 6 peaks fit of XPS spectra of Ti 2p level of W-TiN NPs sample.
